# Investigation of the immune effects of *Scutellaria baicalensis* on blood leukocytes and selected organs of the chicken’s lymphatic system

**DOI:** 10.1186/s40104-017-0152-x

**Published:** 2017-03-01

**Authors:** Bożena Króliczewska, Stanisław Graczyk, Jarosław Króliczewski, Aleksandra Pliszczak-Król, Dorota Miśta, Wojciech Zawadzki

**Affiliations:** 1 Department of Animal Physiology and Biostructure, Faculty of Veterinary Medicine, Wroclaw University of Environmental and Life Sciences, C.K Norwida 31, 50-375 Wrocław, Poland; 2 Department of Immunology, Pathophysiology and Veterinary Preventive Medicine, Faculty of Veterinary Medicine, Wroclaw University of Environmental and Life Sciences, Wrocław, Poland; 3 Department of Chemical Biology, Faculty of Biotechnology, University of Wrocław, Fryderyka Joliot-Curie 14a, 50-383 Wrocław, Poland

**Keywords:** Development of immune organs, Leukocyte, Lymphatic system, Radial segmentation, *Scutellaria baicalensis*, Toxic effect

## Abstract

**Background:**

The health of chickens and the welfare of poultry industry are central to the efforts of addressing global food security. Therefore, it is essential to study chicken immunology to maintain and improve its health and to find novel and sustainable solutions. This paper presents a study on investigation of the effect of *Scutellaria baicalensis* root (SBR) on the immune response of broiler chicken, especially on lymphocytes and heterophils reactivity, regarding their contribution to the development of immunity of the chickens.

**Methods:**

The 121-day-old Hubbard Hi-Y male broiler hybrids were randomly assigned to four treatment groups, three SBR supplemented groups (0.5, 1.0, and 1.5% of SBR) and one control group. Each treatment was replicated five times with six birds per replicate pen in a battery brooder. Blood was collected after 3^rd^ and 6^th^ wk of the experiment, and hemoglobin and hematocrit values were determined, as well as total leukocyte count and differential count were performed. Nitroblue tetrazolium test and phagocytosis assay as nonspecific immune parameters and humoral immune responses to the antigenic challenge by sheep red blood cells were performed. Moreover, the ability of peripheral blood lymphocytes to form radial segmentation (RS) of their nuclei was analyzed. Body weight and relative weight of spleen, liver, and bursa of Fabricius were recorded.

**Results:**

Results showed that mean heterophile/lymphocyte ratio increased in the SBR groups compared to the control group and the blood of the chickens showed lymphocytic depletion. The results also demonstrated that the relative weight of bursa of Fabricius and spleen in groups fed with SBR significantly decreased compared to the control group. This study also showed that the addition of SBR significantly inhibited the formation of RS of nuclei compared to some cytotoxic substances.

**Conclusion:**

We found that SBR supplementation should be carefully evaluated when given to poultry. The excess intake of SBR supplementation may cause immunologic inhibition and may negatively affect the development of immune organs. SBR has inhibited the formation of radial segmentation nuclei showing antimetastatic properties and also the phagocytosis of chicken heterophils.

## Background

In recent years, extensive research has been done on the potential food applications in food products and poultry feeds, for natural antimicrobial agents against foodborne pathogens that improve the health and performance of animals [1]. Novel types of effective and healthy antimicrobial compounds that could protect food and animal against microbiological contamination and the consumer against infection are in high demand. Medicinal herbs, as a new class of additives to animal feeds, can have beneficial properties such as antioxidant, antimicrobial, and antifungal as well as immunomodulatory effects; these properties make them the increasingly used products nowadays [2]. On the other hand, some herbal substances can interact in potentially dangerous ways with the organism systems [3]. Studies have been carried out to investigate the effect of various medicinal plants possessing immunostimulating and antioxidant properties [4], but they focused on the short-term supplementation (<2 wk) to allay the negative effects [5]. Whereas, the nature of commercial feeding programs makes the longer-term efficacy of dietary immune enhancers an important consideration.

Immune response in poultry can be influenced by genetic background, nutrition, environment, and management, or any combination of the above. Chicken heterophils are the first line of defense that can launch a series of intra- and extracellular antimicrobial mechanisms. Improved or reinforced immune response in poultry creates resistance against diseases, may be as the result of preparedness of immune system against pathogenic agents, which is an important factor in improving the homogeneity, long life, growth and the health of a flock. Therefore, greater emphasis has been placed by the researchers on improving the immune response. On this regard, the herb – *Scutellaria baicalensis* (SB) –has received particular attention. However, the effects of other herbs as well as SB on the immune system and selected organs of lymphatic system of chicken are relatively unknown. The lymphoid tissue is involved in the defensive mechanism against microorganisms. The lymphoid system of chicken consists of unique organs and is divided into two morphologically and functionally distinct components: central lymphoid tissue, represented by thymus and bursa of Fabricius and the peripheral lymphoid tissue, represented by the spleen and all mucosa-associated lymphoid tissue [6].

There are practical reasons to study the immune system of poultry, particularly of the chicken. The health and welfare of poultry are central to the efforts of addressing global food security. Over the past two decades, poultry has increased in the world meat production and is still growing. Poultry are also a significant source of zoonotic infections, which can be exemplified by their viruses and bacteria. Antimicrobial compounds (antibiotics) were commonly included in poultry diets for promoting the growth and control the diseases. However, the European Union (EU) banned feed grade antibiotic growth promoters, not only because of the cross-resistance, but also due to the risk of possible multiple drug resistances in human pathogenic bacteria. Thus, the scientific communities have given more attention toward the potential antimicrobial activities of natural products, although using some of them has resulted in decreased body weights, increased feed conversion per kg of weight gain and insignificant effects on carcass yield and carcass fatness. Consequently, from the above reasons, it is therefore essential that we study chicken immunology to maintain and improve poultry health and to find novel and sustainable solutions for the future, because the broilers reach slaughter weight within few weeks. In the EU, the slaughter age ranges from 21 to 170 d, with the average slaughter age of 42 d [7]. This leaves little time to develop immune system. Hence, in the context that the biologically active plant additives could potentially effect or affect the development of mature immune system, we decided to administer chickens with SBR for 42 d with the control in midterm (21 d).

In our previous studies, we showed that the addition of the SBR to fodder at doses of 0.5, 1.0, and 1.5% of SBR did not worsen the quality or chemical composition of the breast and leg muscles of broiler chickens. The addition of 0.5 and 1.0% of SBR in diet had also little effect on performance and measured blood parameters. In the group fed with 1.5% of SBR in diet, the body weight gain, red blood cell count, and hemoglobin level was found to be higher, while the high-density lipoprotein (HDL), low-density lipoprotein (LDL), and total cholesterol levels in blood serum were lower than in the control group [8, 9]. Therefore, in the present study, we chose the same doses of SBR.

The dry root of SB is one of the most widely used Chinese herbal medicines, listed in the Chinese Pharmacopeia [10]. This Asian plant is well acclimatized and cultivated in central European conditions [11]. The dried roots of this plant have a particularly high flavonoid content (over 25%) [10]. The active components of the root of SB have multiple biological properties including anti-inflammatory, antiviral, anticarcinogenic effect, free radical scavenging, and antioxidant effects, as well as antithrombotic and vasoprotective effects [12]. The main flavones of SB include wogonin, wogonoside, baicalin, and baicalein with ratios to the dry material of about 1.3, 3.55, 5.41, and 12.11%, respectively [10], but its content depend on the growing conditions and isolation methods. List of chemical compounds isolated from SB are listed in Dr. Duke’s Phytochemical and Ethnobotanical Database [13].

This study was carried out to investigate the effect of SBR on the immune response of chicken especially on the reactivity of lymphocytes and heterophils, regarding their contribution to the development of immunity. Moreover, a phagocytic test was performed to evaluate the ability of heterophils to ingest yeast cells and to furthermore evaluate the ability of peripheral blood lymphocytes to form RS of their nuclei. At the same time, the lymphocyte cytoskeleton status was evaluated indirectly by inducing the RS of lymphocyte nucleus to determine whether SBR affects the leukocyte function of both groups in the same way.

## Methods

### Plant material

Baikal skullcap, *Scutellaria baicalensis* Georgi, plants were grown in the University’s experimental field from authenticated seeds obtained from the botanical garden of the medicinal plant herbarium at the Wroclaw Medical University, Poland. Voucher specimens were deposited at the herbarium. In the spring, the seeds were sowed in light well-drained sandy soil in partial shade. Plants were watered weekly as needed. Fertilizers were supplied along with the water over the growing season via the drip tape to provide 0.012, 0.008, and 0.01 g/m^2^ of N, P_2_O_5_, and K_2_O, respectively. The roots were harvested in autumn from 2-year-old plants. The roots were collected, thoroughly, washed in distilled water, and dried under controlled humidity of 25 °C until a moisture content of 5% was reached [14]. The dried roots were then crushed using a laboratory mill and stored at −20 °C until use.

### Analytical HPLC analysis of flavonoids

High-performance liquid chromatography (HPLC) was used for the analytical determination of flavonoids in SB. Samples and standards were analyzed using a Waters 600 system coupled to Waters 2487 UV dual wavelengths absorbance detector (Waters Chromatography Canada Inc.), and Empower PDA software (Milford, MA USA). An Agilent Zorbax SB-C18 column (4.6 × 250 mm, 5 μm) was applied for this analysis. Sample preparation and extraction of flavonoids were done using a previously described method with modifications [15]. Before extraction, the herbal powder was soaked in Britton-Robinson buffer, pH 6.5 at 50 °C for 30 min. Flavone standards, baicalin (99%), baicalein (98%), wogonoside (≥95%), wogonin (≥98%) were purchased from Sigma-Aldrich. All HPLC-grade solvents were filtered through a membrane filter (0.2 μm pore size). The content of the constituents was calculated using standard curves acquired for four flavonoids. All measurements were performed in triplicate.

### Animals, diets, and experimental design

One hundred and twenty one-day-old Hubbard Hi-Y male broiler hybrids were vaccinated against Newcastle Disease and Infectious Bronchitis. Vaccines were delivered via spraying method. No other vaccination was performed during the experiment. One-day-old broiler chickens with a mean body weight of 39 g (±1.7 g) were randomly assigned in four treatment groups. Each treatment was replicated five times with six birds per replicate pen in a battery brooder. All the pens were equipped with feeders and water. The birds were fed a starter diet for 21 d, followed by a finishing (grower) diet from d 21 to d 42. The basal diets were formulated based on NRC (National Research Council) guidelines and contained 18.50–20.10% crude protein and 12.13–12.55 MJ/kg metabolizable energy [16]. The composition, preparation, and suitability of the experimental diets were followed as described in previous study [8]. Chemical analysis of the principal components in diet was performed by standard methods as described in the Association of Official Analytical Chemists [17]. The chicks were housed in electrically heated battery pens, and the diets and fresh water were provided ad libitum. The birds were fed either a basal diet or a diet supplemented with ground and dried SBR. The experiment included three groups supplemented with SBR (0.5, 1.0, and 1.5% of SBR) and one control group (C) with no supplementation of SBR.

### Performance data

Body weight, feed intake and chicken mortality were determined. All the chickens in each pen were weighed in groups at the beginning and at the end of the experiment. Weight gain was obtained from these data. The feed consumed per pen was recorded; average daily feed intake (ADFI) and feed conversion ratio (FCR) from d 0 to d 42 were calculated.

### Hematological parameters

Blood was collected after wk 5 and wk 6 of the experiment. Blood was taken from the brachial vein of six chicken chosen randomly from each treatment group. Hemoglobin (HGB) and hematocrit values (HCT) were determined. The HGB was determined spectrophotometrically [18] and HCT was determined by centrifuging the blood in glass capillaries at 10,000 × g for 5 min. One drop of blood from each sample was smeared on a glass slide. The smears were stained by May-Grünwald-Giemsa staining method (MGG) [19]. The smears were used to perform leukogram, by counting up to 200 leukocytes. The percentage of leukocytes (WBC) including heterophils, lymphocytes, basophils, eosinophils, and monocytes were determined by counting up to 200 cells. The heterophils/lymphocytes ratio (H/L) was calculated as well.

### Immunization

At 5 wk of age, six chicks from each treatment group were injected intravenously in the brachial vein with 0.5 mL of 10% suspension containing 1 × 10^8^/mL packed sheep red blood cells (SRBCs) (Sigma-Aldrich) in PBS (phosphate buffered saline). Blood samples were taken from brachial vein 7 d after immunization (at 6 wk of age). Sera were obtained by centrifuging blood at 3500 × *g* for 15 min. To determine the antibody response to SRBCs, a direct hemagglutination assay was used and the total antibody (IgM and IgY) response to SRBCs in serum was measured. At first, to inactivate the complement proteins, serum samples were incubated for 30 min at 56 °C. To inactivate IgM component and measure anti-SRBC antibodies (IgY), serum samples were mixed with equal volume of 0.2 mol/L 2-mercaptoethanol for 30 min at 37 °C. Then the serum samples were serially diluted with PBS in 2-fold steps (1:1 – 1:1012) in U-bottomed microplates (96 well, Medlab, Poland), 100 μL/well. In the next step, 25 μL of 2% SRBC suspension was added to each well. The plates were incubated for 18 h at 37 °C. The titer of the well containing 50% SRBCs agglutination was recorded as positive result. The “titer” is defined as the reciprocal of the serum dilution that has an optical density (OD) of 0.5. If a serum has an OD value of ≥ 0.5, the reciprocal of the starting serum dilution that is closest to an OD value of 0.5 will be used as the titer. The serum titer is converted to log_2_ titer and the log_2_ titer is recorded in thousand units. The log_2_ titer data may be calculated [20] using the following formula.

$$ { \log}_2\ \mathrm{titer}=\frac{{ \log}_{10}\ \left(\mathrm{titer}\right)}{0.301} $$

If a serum does not meet the criteria as described above, then the serum may be tested again using a starting dilution, which may be either higher or lower than the previous starting dilution, or the data for that particular serum may be excluded.

### Radial segmentation of lymphocyte nuclei

Blood samples were used to test the RS of lymphocyte nuclei with some modification [21]. The collected blood samples were divided into two equal parts of 0.8 mL. Subsequently, 0.2 mL of oxalates mixture (a solution of 0.57% potassium oxalate and 0.85% ammonium oxalate mixed in a ratio of 1:1) was added to one part of the 0.8 mL heparinized blood (RS induced). The second one without oxalate was used as a control (RS spontaneous). Both the samples were incubated for 3 h at 21–23 °C. Later, the samples were centrifuged (1500 × g, 10 min), the leukocyte layer situated between erythrocytes and plasma was collected, and then three smears from each chick were prepared. The smears were fixed in methanol and stained according to Pappenheim’s panoptic method with MGG stain. The steps that followed involved the differentiation between RS positive (RS+) and RS negative (RS–) lymphocytes (the lymphocytes found were counted up to 200), according to the criteria described in previous study [22]. Those cells whose nuclei have slots with the depth of at least one-third of its diameter were considered RS+. The results of the RS test obtained were in a medium range for each group.

### Phagocytosis assay

Phagocytosis assay based on the method of Pliszczak-Król et al. [23] was performed. *Saccharomyces cerevisiae* cells were used to evaluate the phagocytic potential of heterophils. The mixture of heat-inactivated yeast cells (100 μL, 8 in McFarland’s scale) and heparinized blood (1 mL) was incubated for 15 min at 37 °C. After incubation, two smears were prepared from each blood sample, air dried, and stained using MGG. The smears were analyzed up to 200 granulocytes, differentiating them as positive phage (Fag+) and negative phage (Fag–). The percentage of phagocytosis by heterophils was then calculated.

### Nitroblue tetrazolium assay

The bactericidal activity of phagocytic cells was measured based on the method described by Chung and Secombes [24]. Nitroblue tetrazolium (NBT) reduction assay was performed by spectrophotometric method. A total of 0.1 mL of blood sample was added to 0.1 mL of 1% solution of NBT; control sample with 0.1 mL of PBS instead of NBT solution was prepared, and the samples were then incubated for 30 min at 37 °C and again for 30 min at room temperature for formazan to be formed. Then, 0.05 mL of sample was taken and the blue formazan product formed was dissolved by adding 1 mL of dimethyl sulfoxide (DMSO). The sample was centrifuged (3000 × g, 5 min) and the absorbance of the supernatant was measured at a wavelength of 560 nm.

### Determination of body weights and relative weights of immune organs

At the end of the experiment, six chicks from every experimental group were randomly chosen to be weighed and slaughtered. The chicks were individually weighed before slaughter. They were sacrificed by decapitation and left to complete bleeding. The liver, bursa of Fabricius, and spleen were removed and weighed. The body weight (BW) of each chick in grams was determined. On this basis, the relative weight (RW) of all organs was calculated, e.g., the RW of immune organ is equal to the weight of immune organ minus the chicken’s body weight.

### Statistical analysis

The Shapiro-Wilk or D’Agostino-Pearson normality tests were used to analyze the normally distributed population. The Shapiro-Wilk test works very well if every value is unique, whereas it is not as effective when several values are identical. In those cases, the D’Agostino-Pearson test was used. Statistical analyzes of variance were calculated using ANOVA followed by a *post-hoc* test (Bonferroni multiple comparison test). Multiple comparisons were done only when the ANOVA *P*-values were significant. The *P*-value was calculated under the null hypothesis that the samples were drawn from the same distribution. The *P* < 0.05 were considered statistically significant. The effects of the SBR were estimated by analyzing the results of the eight lymphoid organs and SRBC antibody titer by polynomial regression model. Pearson correlation coefficient (r) with two-tailed test of significance was conducted to examine the relationship between certain parameters. Analyzes were performed with Statistica version 10.0 (StatSoft Inc., Tulsa, OK, USA).

## Results

### Growth performance

The results for the production traits of chickens fed with and without SBR in diet are presented in Table 1.

**Table 1:** Growth performance of chickens fed with experimental diets from 1 to 42 d of age^d^

Parameter	Dietary treatments			
	Control	0.5%	1.0%	1.5%
BW, g				
d 1–42	1549 ± 22^a^	1432 ± 39^b^	1626 ± 24^c^	1589 ± 28^c^
BWG, g				
d 1–42	1509 ± 29^a^	1394 ± 17^b^	1585 ± 26^c^	1549 ± 32^c^
ADFI, g/d				
d 1–42	70.83 ± 1.23^a^	71.02 ± 1.30^a^	74.54 ± 1.19^b^	75.38 ± 1.28^b^
FCR, g/g				
d 1–42	1.97^a^	2.12^b^	1.98^a^	2.04^a^

^a,b,c^Mean values within the same row not sharing a common uppercase superscript letter differ significantly (*P* ≤ 0.05)

^d^Body weight (BW; g), body weight gain (BWG), average daily feed intake (ADFI; g), and feed conversion ratio (FCR; g/g) averaged over treatment period for chicken (from 1 to 42 d of age)

The results show that dietary SBR did not affect body weight. No mortality rate was recorded for broiler chickens fed with SBR diet for the whole experimental period. The addition of SBR to the chicken fodder has affected the final body weight gain of the birds. On the 42 d of breeding, the final weights of the chickens in the experimental groups were higher than the final weight of the control group chickens: higher by 4.9% for the group with a 1.0% supplement, higher by 2.6% for the 1.5% group and it is lower by about 7.5% for the 0.5% group (*P* < 0.05). We showed that in broiler chickens fed with SBR supplemented diet, the ADFI was increased (*P* < 0.05) in groups supplemented with higher doses of SBR (1.0 and 1.5%). The increase in FCR was up to 7% significantly higher in the experimental group receiving the fodder with the 0.5% concentration of SBR.

### Quantitative analysis of the flavonoids

The quantitation of baicalin, baicalein, and wogonin was achieved using peak area ratios of baicalin, baicalein, wogonin, and wogonoside to the internal standard. The amount (mg/g of dry weight) of baicalin, baicalein, and wogonin in the SBR is presented in Table 2.

**Table 2:** The concentration of baicalin, baicalein, and wogonin present in dry roots of *Scutellaria baicalensis* ^a^

Flavonoids	Concentration, mg/g DW^a^	Total flavonoids concentration, mg/g DW^b^
Baicalin	153.2 ± 8.1	214.5 ± 13.3
Baicalein	19.3 ± 2.3	
Wogonin	8.2 ± 1.2	
Wogonoside	29.8 ± 1.7	

^a^All values are mean ± SD (*n* = 3)

^b^dry weight

Total flavonoids content was 214.5 ± 13.3 mg/g of dry weight (DW). The chemical profile of the SBR extract was obtained with a constant ratio of 5:1 for baicalin/wogonoside. Due to poor solubility of baicalin and baicalein in water, its absolute bioavailability after oral administration is 2.2 and 27.8%, respectively [25]. During our study, chickens adsorbed baicalin at doses of 120.42 mg/d, 251.22 mg/d, and 386.14 mg/d in group receiving 0.5, 1, and 1.5% of SBR, respectively.

### Blood hematology

**Fig. 1:**
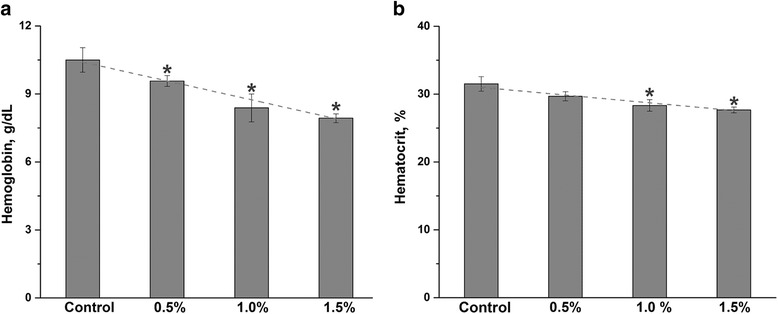
The effect of SBR diet on RBC. Hemoglobin **a**, and hematocrit **b** were measured in chicken fed SBR containing either 0.5%, 1.0% or 1.5% SBR. The SBR caused a statistically significant (*) reduction in both blood parameters in chicks fed with SBR (*P* < 0.05). Dash line shows the linear relationship between the SBR concentration and HGB or HCT levels. The results are reported as a mean ± SD (*n =* 6)

The HGB and HCT contents have been significantly (*P* < 0.05) decreased in group receiving diets supplemented with SBR at d 42 of feeding (Fig. 1). A linear decrease in the HGB and HCT was observed with increasing SBR dose. The Pearson’s correlation coefficient (r) for these data was −0.995 and −0.980, respectively (*P* < 0.001). The effect of SBR diet at various percentages on particular type of leukocytes is summarized in Table 3.

**Table 3:** Effect of experimental diets on the percentage of particular types of leukocytes at d 42, (*n* = 6)^d^

Parameter	Dietary treatments				SEM	*P*-value	r^*^
	Control	0.5%	1.0%	1.5%			
WBC, 10^3^/μL	3.16^a^	3.07^a^	2.98^b^	2.34^c^	0.49	0.050	−0.884
Lymphocytes, %	63^a^	56.33^b^	53.33^b^	48.83^bc^	5.35	0.048	−0.989
Heterophils, %	30.5^a^	38^b^	39^b^	45.83^b^	6.12	0.032	0.930
Eosinophils, %	3.50^a^	2.33^b^	0.83^c^	1.83^b^	1.19	0.044	−0.757
Basophils, %	2.33	3.17	3.16	2.83	1.12	0.864	0.177
Monocytes, %	0.66	0.16	0.66	0.66	0.38	0.483	0.199
H/L ratio	0.51^a^	0.71^b^	0.73^b^	1.01^b^	0.19	0.043	0.959

^a,b,c^Means in the same row with different superscripts are significantly different (*P* < 0.05)

^d^The results are expressed as the mean for 6 birds and 2 repeats. Control = basal diet; 0.5% = basal diet + 0.5% of SBR, 1.0% = basal diet + 1.0% of SBR, 1.5% = basal diet + 1.5% of SBR

*Pearson’s correlation coefficient

Total blood WBC, total lymphocyte, heterophils, and eosinophils counts on d 42 differed markedly among treatments (*P* < 0.05). On d 42, a decrease in WBC counts (*P* < 0.05), which included lymphocytes and eosinophils, was found (Table 3). The Pearson’s correlation coefficient showed significant (*P* < 0.01) decreasing relationship between SBR concentration and WBC or lymphocytes or eosinophils count.

Significantly higher amounts of heterophils and H/L ratio were determined in chicken fed graded levels of SBR compared to the control group (Table 3). These studies have demonstrated that the Pearson’s correlation coefficient showed significant (*P* < 0.01) increasing relationship between SBR concentration and amounts of heterophils, and hence also higher H/L ratio. Monocytes and basophils count did not differ significantly (*P* < 0.05) between the experimental groups, and no correlation was observed.

### Phagocytosis assay

**Fig. 2:**
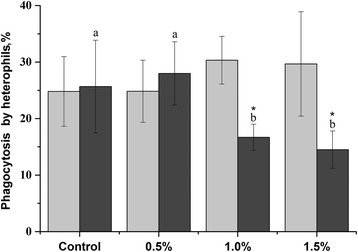
Percentage of phagocytosis of *Saccharomyces cerevisiae* cells by heterophils. Heterophils were isolated from the blood of broiler chickens fed with graded concentrations of dietary SBR. Different letters indicate the significant difference (*P* < 0.05) among the groups, * indicates significant differences on d 21 and d 42. Light gray—d 21, dark gray—d 42. The results are reported as a mean ± SD (*n =* 6) from duplicate samples

The effect of graded levels of dietary SBR on the percentage of heterophils phagocytizing *Saccharomyces cerevisiae* cells is shown in Fig. 2. The inhibition of phagocytosis was measured after d 21 and d 42 of feeding with SBR diet. Statistical analysis has revealed that on d 42 SBR diet of 1.0 and 1.5% decreased (*P* < 0.05) the percentage of phagocytic activity of heterophils significantly (16.7 and 14.5%, respectively). Lower level of SBR diet (0.5%) did not inhibit phagocytosis and there is no difference between d 21 and d 42.

### Superoxide anion production/NBT assay

**Fig. 3:**
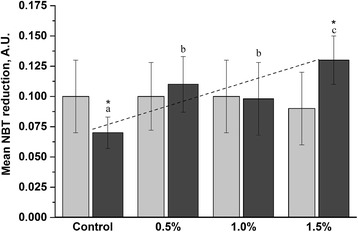
The blood activity as measured by NBT assay. Activity measured in chicken fed with SBR for 21 d (*light gray*) and 42 d (*dark gray*). All the results were calculated on the estimation of 1000 leukocytes. Different letters indicate significant difference (*P* < 0.05) among the groups at d 21 and d 42. * indicates significant differences between d 21 and d 42 among groups. Dash line shows linear relationship between the SBR concentration and mean NBT on d 42. The results are reported as a mean ± SD (*n =* 6) from duplicate samples

The oxidative radical production in blood was measured by NBT assay. No significant difference (*P* > 0.05) was observed in any other feed-treated groups on d 21 compared to the control group (Fig. 3). There was a significant difference (*P* < 0.05) for the NBT activity between the control and all treatment groups d 42 (Fig. 3). The mean OD values for the heterophils of treatment groups at d 42 (0.5, 1.0, and 1.5%) were found to be 0.117 ± 0.06, 0.098 ± 0.03, and 0.128 ± 0.02, respectively, while for the control group it was 0.068 ± 0.04. Statistical analysis done by NBT assay revealed that at d 42 SBR diet of 1.5% has increased (*P* < 0.05) the superoxide anion production significantly.

Moreover, a positive tendency (*r* = 0.93), reflected by increased immunological indices (NBT reduction), was recorded in the group fed with SBR supplements at the end of the experimental period (d 42).

### Humoral antibody response to SRBC

**Fig. 4:**
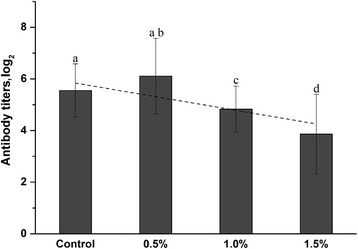
Changes in anti-SRBC post-vaccine immune response in chicken fed with SBR. Dash line shows the linear relationship between the SBR concentration and antibody titers of immunized groups. The results are reported as a mean ± SD (*n =* 6) from duplicate samples. Different letters indicate statistically significant differences between groups (*P* < 0.05)

We evaluated the effects of SBR on the humoral antibody-mediated responses to SRBCs. SRBCs are natural nonpathogenic antigens, which stimulate a wide range of immune cells with their multiple antigen binding sites after immunization with SRBCs [26]. Immunization with SRBCs resulted in the rise of specific anti-SRBC antibody in serum (Fig. 4). Antibody response against SRBC was detected in all groups. There was a statistically significant (*P* < 0.05) linear decrease (*r* = −0.82) in the antibody titers over immunized groups. The anti-SRBC antibody titers at wk 1 post-immunization in the SBR-treated groups were 6.16, 4.83, and 3.86, respectively. In the control group, the observed level was 5.45. As seen in Fig. 4, there was a consistent trend in SRBCs titer, but the level of anti-SRBC in the group treated with 0.5% of SBR diet was higher (*P* < 0.05) as opposed to the other SBR groups. However, there was not any significant difference between control and 0.05% groups. The antibody response to SRBC was significantly lower (*P* < 0.05) in the group fed with 1.0%, in contrast to control and 0.5% groups. Particularly, we observe a more marked decrease (*P* < 0.01) in the group fed 1.5% SBR.

### RS of blood lymphocytes nuclei formation

The influence of SBR on the rate of formation of RS nuclei was studied. The ability to form RS was estimated in parallel to the leukogram analysis [27]. The aim of the experiment was to evaluate the ability of peripheral blood lymphocytes isolated from chicken blood to form the RS of their nuclei. The term radial segmentation (RS) is applied to a characteristic nuclear deformation, occurring in vivo in many neoplastic and leukemic cells, but often also as an in vitro artifact in different conditions [28]. Furthermore, the RS phenomenon is more frequent in infectious, inflammatory, and necrotizing diseases than in healthy subjects [29]. The normal segmentation of the nucleus is quite different from RS due to the pathologic or toxic environment, for example, storage in a culture medium with in EDTA sodium oxalate.

**Fig. 5:**
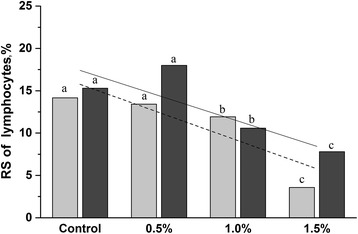
The percentage of lymphocyte nuclei undergoing RS in experimental groups. Light gray—d 21, dark gray—d 42, showing differences among the groups on d 21 and d 42. Dash line show linear relationship (*r* = −0.87) between the SBR concentration and RS formation on d 21, solid line shows linear relationship (*r* = −0.94) on d 42. Different letters indicate statistically significant differences among groups (*P* < 0.05). Values represent means ± SD (*n*  =  6) from duplicate samples

Radial Segment formation was determined at d 21 and d 42 stage. The levels of RS+ in control group were 14.7 and 15.5, respectively. The percentage of RS+ cells changed nonsignificantly in the chicken fed with 0.5% of SBR. In the groups fed with 1.0 and 1.5% SBR, the percentage of RS+ cells decreased significantly (*P* < 0.01) in both the weeks tested (Fig. 5). However, a two-fold decrease in the percentage of RS+ cells was observed in birds fed with 1.5% of SBR compared to the control group on d 21 and d 42 (3.58 and 7.80, respectively, Fig. 5).

### Relative weight of selected organs

Addition of SBR in the diet did not influence (*P* > 0.05) absolute or relative weight (RW) of the liver on d 42; however, there was a linear decrease (*P* < 0.05) in the RW of spleen and bursa of Fabricius when the birds were 42-day-old (Table 4).

**Table 4:** Morphometry of lymphoid organs of broilers fed with diets containing different amounts of SBR, (*n* = 6)^c^

Parameter	Dietary treatments				SEM	*P*-value	r^*^
	C	0.5%	1.0%	1.5%			
Liver RW, mg/100 g BW	1.97	2.09	2.06	1.78	0.12	0.087	−0.25
Bursa of Fabricius RW, mg/100 g BW	282.70^a^	284.46^a^	232.60^b^	215.72^b^	38.28	0.050	−0.95
Spleen RW, mg/100 g BW	169.63^a^	145.50^b^	136.90^b^	141.82^b^	25.73	0.043	−0.79

^a,b^Means in the same row with different superscripts are significantly different (*P* < 0.05)

^c^The results are expressed as the mean for 6 birds and 2 repeats. Control = basal diet; 0.5% = basal diet + 0.5% of SBR, 1.0% = basal diet + 1.0% of SBR, 1.5% = basal diet + 1.5% of SBR

*Pearson’s correlation coefficient

The calculated RW of the spleen decreased by 14.22, 19.29, 16.39%, respectively, compared to the control group (*P* < 0.05). Similar results were observed for the RW of bursa of Fabricius. However, RW nonsignificantly increased in group fed with 0.5% SBR. The RW decreased by 17.72 and 23.69% in groups fed with 1.0 and 1.5% of SBR, respectively, compared to control group (*P* < 0.05). A linear decrease in the RW of spleen and bursa of Fabricius was observed with increasing SBR dose (*r* = −0.95 and −0.79 respectively).

## Discussion

Baicalin has been safely used for the treatment in several animal models of diseases and pharmacokinetic properties of baicalin have been well investigated [30]. Both oral administration and IP injection was used for baicalin treatment at a wide range of dosage (10 ~ 800 mg/kg/d) in various animal models [30]. Baicalin and baicalein showed weak absolute bioavailability after oral administration [25]. Similar or lower levels of bioavailability of flavonoids are observed in SBR orally administered animal models [31]. This was the second reason why we chose doses in the range of 0.5–1.5%, which provided average level of flavonoids used in other studies [30].

The presented results concerning chickens fed with supplementary SBR were positive with regard to bird breeding parameters. The data from this study also indicates a trend toward the ADFI of broiler chickens receiving the SBR in their diet (*r* = 0.97). Additional studies are needed to explain growth performance results of the group supplemented with 0.5% SBR, especially concerning body weight (BW).

Evaluation of hematological parameters usually provides significant information on the body’s response to injury, they are a good indicator of the physiological and health status of animals and they can be useful to complement the knowledge on unfamiliar effect of feed additives [32].

In this study, HGB and HCT values were significantly lower (*P* < 0.05) in birds that received diets supplemented with SBR. Despite, these levels could be framed within the reference intervals provided by Bounous and Stedman [33]. However, significant (*P* < 0.001) linear decrease for both the parameters was observed during experiment with increasing dose. The values of WBC, heterophils, basophils, and lymphocytes levels were within the physiological limits found in the reference of previous studies, while the percentages of monocytes and eosinophils were lower in all groups [34]. However, even in these cases, we observed a significant (*P* < 0.05) linear change with increasing dose of SBR. In general, the blood of the chickens showed lymphocytic depletion, which may represent a combination of direct effects of SBR components and/or nonspecific stress factors.

The H/L ratio have been widely accepted in many disciplines of avian research as a measure of the chicken’s perception of stress in its environment [35] and is useful in assessing the efficiency of the immune system and health condition of birds [36]. The H/L proportion and ratio were found to be significantly increased (*P* < 0.01) in the SBR groups compared to the control group. However, growth of heterophils could be the consequence of mobilization of the immune system to phagocytosis. A number of in vitro and animal studies have shown that bioactive compounds from plants increase immunologic activity by increasing phagocytosis [37]. Phagocytosis by granulocytes is the first and major defense mechanism against invasion of bacteria, fungi, and parasites.

Results from the present study demonstrate the inhibition of phagocytosis by heterophils when chickens were fed with medium (1.0%) and high (1.5%) doses of SBR. Similar findings have been recorded in fish by Yin et al. [38] and in mouse by Cai et al. [39]. However, in contrast to our study these researchers have used only baicalin.

Degranulation and production of oxidative burst are closely associated with phagocytosis [40]. In this process, mediated by a multicomponent enzyme complex, nicotinamide adenine dinucleotide phosphate oxidase (NADPH-ox) phagocytic heterophils consume oxygen and produce reactive oxygen species (ROS) [40].

This study demonstrates a significant increase (*P* < 0.05) in the capacity of these cells to reduce NBT on d 42 compared to d 21 (Fig. 2). Therefore, an increased level of active toxic oxygen compounds may suggest a greater efficiency of oxidative response [41]. Flavonoids from SBR, such as baicalin or baicalein, to be a very effective inhibitor of the production of ROS by human leukocytes [42]. In addition to the inhibitory action on the production of ROS, both baicalein and baicalin showed a strong eliminating activity toward · O_2_^−^, but showed no significant effect on scavenging hydroxyl radical (·OH) [43].

Many functional and morphological changes are observed during lymphocyte activation in which a major role is ascribed to cytoskeletal microtubules and include the numerous processes occurring in cells. These changes take place in both mature and maturing leukocytes. The similar pattern of functional and morphological changes was observed in lymphocytes activated by mitogens or in neoplastic ones [44].

For the evaluation of cytoskeletal lymphocytes, the indirect test of RS formation was used. Radial segmentation is a fascinating phenomenon, yet to be fully explained, that involves the leucocyte nuclei of various diseases [29]. RS of nuclei may provide a convenient and reliable screening test for metaphase-blocking activities of new substances [45]. Söderström et al. [45] indicated a relation between the ability of lymphocyte nuclei to deform and the reorganization of cytoskeletal elements, including tubulin, filaments, and actin.

This study has showed that the addition of 1.0 and 1.5% SBR significantly (*P* < 0.05) inhibited the RS nuclei formation. The cytochalasin B possessing cytotoxic activity show such effects [46]. Thus, cytochalasin B converts neutrophils from phagocytic cells into model secretory cell [47]. There is no guarantee that the same mechanism or process is activated by SBR, but the inhibition of phagocytosis and increase in the capacity of these cells to reduce NBT indicate the possibility of similar modes of action. Kumagai et al. [48] showed that SB inhibits the proliferation of lymphocytic leukemia, lymphoma, and myeloma cell lines by the induction of apoptosis.

Birds have central and peripheral lymphoid tissues, which play an important role in the body defense against pathogens [6]. The thymus and bursa of Fabricius are the primary lymphoid organs, whereas secondary lymphoid organs include spleen and all the lymphoid tissues associated to the intestinal mucosa. The spleen is the major site of immune responses to blood-borne antigens and is also a site of hematopoiesis [49].

This study was aimed at elucidating the effects of SBR on the immune responses in chickens. SRBC antigens elicit antibody-mediated responses in chickens [50]. Immunized chicken showed a significantly lower (*P* < 0.05) antibody response to SRBCs, when treated with SBR (1.0 and 1.5%). In the group supplemented with 0.5% of SBR, we observed slight, but not significant, increase in antibody response. Different result from the studies on mice was reported by Jong et al. [51]. The antibody response to T lymphocyte-dependent antigen, (SRBC) was increased at all doses (100, 200, and 400 mg/kg) of SB-treated mouse.

The mechanisms of the weakness of immune responsiveness conferred by SBR in our studies require further investigation.

Considering the RW of organs, only the liver showed similar weight for 42-day-old birds of all the groups receiving SBR diet. In this study, the body weight and RW were used for the evaluation of the state of the development of the spleen, which is responsible for initiating immune reactions. The researchers reported that at 42 d of age, the weight and RW of the spleen and bursa of Fabricius of birds fed with SBR was found to be low and that SBR can inhibit the development of the spleen. Moreover, it was observed that lymphopenia appeared in all SBR-fed groups, which could be a reason for the decreasing weight of spleen, and a possibility that SBR could affect lymphocyte proliferation by changing the production of cyclins and interleukins responsible for their proliferation [52]. Baicalin significantly decreased the production of IL-10. According to the cancer immunoediting theory inhibition of the protective functions of the immune system via overproduction of immunosuppressive cytokines, such as IL-10, may also facilitate tumor escape [53]. Yang et al. [54] reported that baicalin, is responsible for the inhibition of the synthesis of inflammatory mediators such as IL-6 and differentiation of T_H_17 cells in spleen. Intraperitoneal administration of baicalein (100 mg/kg) enhanced apoptosis of T and B cells in spleens [55]. Baicalin also could promote T regulatory cell differentiation and upregulate the function of T regulatory cells and may also serve as a natural immunosuppressive compound for treating autoimmune inflammatory diseases [54].

The bursa of Fabricius is responsible for the establishment and maintenance of the B-cell compartment in birds [56]. The most rapid growth of bursa occurs in the first 3–5 wk of life and reaches its maximum size in 4 to 12 wk of age [57]. The RW of the bursa did not decrease in group that was fed with 0.5% of SBR diet. However, the results indicated that excess SBR intake (≥1%) decreased the growth of the bursa in young chicken. The lymphopenia could also be a reason for the decreased weight of the bursa of Fabricius. Atrophy of the bursa due to lymphocytic depletion is also caused by viral diseases, bacterial diseases, mycotoxicosis, nutritional diseases, immunosuppressive drugs such as cyclophosphamide, and chick anemia agents [58].

## Conclusion

Antibiotics have played a negative role in the animal industry due to the hidden danger of drug residues to human health. In the past two decades, an intensive amount of research has been focused on the development of alternatives to antibiotics. Chinese herbal medicines like *S. baicalensis* contain the bioactive components, which possess antibacterial and anti-inflammatory properties. Current evidence strongly suggests that SBR supplementation in the diet of chicken should be carefully evaluated, as only a narrow dose range of SBR could be considered safe for the poultry. Dietary SBR supplementation in excess of 1% affected the humoral immunity of chickens and restrained the development of immune organs in birds. The exact mechanism that mediates the immune response observed in this study is not fully understood, because SBR has a wide variety of effects and requires further investigation. These findings may provide with some information to researchers and feed producers in planning the future diets for poultry. Moreover, the effect of SB is dose-dependent and there is always a potential for overdosing consequently; hence, dosage optimization is strongly recommended. Therefore, these results suggest that further research is required in this area and that the perfect antibiotics alternative does not exist as yet.

## Abbreviations

ADFI: Average daily feed intake
BA: Baicalin
DW: Dry weight
FCR: Feed conversion ratio
H/L: Heterophils/lymphocytes ratio
HCT: Haematocrit
HGB: Hemoglobin
HPLC: High-performance liquid chromatography
MGG: May-Grünwald-Giemsa staining method
NBT: Nitroblue tetrazolium
OD: Optical density
ROS: Reactive oxygen species
RS: Radial segmentation
RW: Relative weight
SB: *Scutellaria baicalensis*
SBR: *Scutellaria baicalensis* root
SRBCs: Sheep red blood cells
WBC: Leukocytes
